# Recombinant Adenovirus-Mediated Intestinal Trefoil Factor Gene Therapy for Burn-Induced Intestinal Mucosal Injury

**DOI:** 10.1371/journal.pone.0062429

**Published:** 2013-04-24

**Authors:** Yong Sun, Yun Zhu, Liangxi Wang, Xuefei Mao, Xi Peng, Yizhi Peng

**Affiliations:** 1 Department of Burn Surgery, Plastic Surgery Center, No. 97 Hospital of PLA, Xuzhou, China; 2 Department of Gastroenterology, No. 97 Hospital of PLA, Xuzhou, China; 3 State Key Laboratory of Trauma, Burns and Combined Injury, Institute of Burn Research, Southwest Hospital, The Third Military Medical University, Chongqing, China; University of Michigan School of Medicine, United States of America

## Abstract

Intestinal trefoil factor (ITF) is a small polypeptide with potential medical values whose main pharmacological effects are to alleviate gastrointestinal mucosal injury caused by various injury factors and promote the repair of damaged mucosa. However, its low yield limits its application. The purpose of our study was to construct a recombinant adenoviral vector containing the *hITF* gene and observe the therapeutic effect of burn-induced intestinal mucosal injury using *in vitro* and *in vivo* analysis. First, a recombinant shuttle plasmid was constructed by ligating a pAdTrack-CMV vector with a full-length *hITF* gene containing a signal peptide and the mature peptide, followed by the recombinant Ad-hITF adenovirus vector after linearization and homologous recombination with the backbone plasmid in the competent BJ5183 strain. Second, the hITF expression level was detected using reverse transcription polymerase chain reaction and western blotting after Ad-hITF infection of colon cancer HT-29 cells. The recombinant adenovirus significantly promoted cell migration in an *in vitro* wounding model. Finally, we confirmed that the recombinant adenovirus could significantly expedite the healing of intestinal mucosal injury after establishing a mouse model in which severe burns were stimulated and the recombinant adenovirus was delivered by intragastric injection. In summary, we constructed a recombinant adenoviral vector containing the *hITF* gene and confirmed its role in promoting repair of the intestinal mucosa. Our study provides a novel way to treat burn-induced intestinal mucosal injury.

## Introduction

Intestinal trefoil factor (ITF), a small polypeptide secreted by intestinal goblet cells [1], [2], was named for its characteristic “trefoil” disulphide loop composed of a special core domain in its 59-amino-acid sequence. The core domain contains 6 cysteine residues linked by disulfide bonds in specific positions and thus forms 3 cyclic structures that comprise a trefoil-like structure after bending and folding of the entire peptide chain [3]. Once discovered, ITF was widely studied by many scholars, and a number of reports have shown that ITF plays an important role in intestinal mucosa self-protection and repair [4], [5]. However, as a pro-drug with broad use prospects, its complex preparation process and low yield limit its application. Gene therapy, a newly emerged means of treatment, treats diseases by delivering normal human gene or gene with a therapeutic effect into human target cells in a certain way to correct genetic defects or play a therapeutic role [6], [7]. If ITF can be delivered into human body by gene therapy and then abundantly expressed *in vivo*, it will both simplify the tedious preparation procedure and reduce production costs.

The core and key of gene therapy is to select a safe and efficient carrier for injecting the gene into cells and then effectively controlling its expression [8]. Gene delivery carriers are either non-virus or virus vectors. The infection efficiency of a viral vector is much higher than that of a non-virus vector, and as one of the most widely used viral vector, the advantages of adenovirus include its wide host range, safety, high titer, multi-gene expression, stability, large capacity, and high exogenous gene expression level, ability to infect both proliferating and non-proliferating cells, human gene homology, and easy immune pathway [9], [10]. Thus, we intend to select the adenovirus as a gene delivery carrier to mediate *hITF* gene expression and observe the protective effect of *hITF* gene therapy on the intestinal mucosa after burn to lay the foundations of clinical application of hITF as a new type of gastrointestinal mucosal protective agent.

## Materials and Methods

### 1. Reagents, Plasmids, Strains and Cell Lines

The protocols in this study were approved by institutional review board and the Animal Care and Use Committee of No. 97 Hospital of PLA (Xuzhou, China). T4 DNA ligase and restriction endonucleases *Bgl*II and *Not*I were purchased from TaKaRa (Dalian, China). Restriction endonucleases *Pme*I and *Pac*I were obtained from Biolab (Lawrenceville, GA, USA). Mouse anti-hITF antibodies and goat anti-mouse horseradish peroxidase-labeled secondary antibodies were purchased from Abcom (Cambridge, MA, USA). Lipofectamine 2000 was obtained from Invitrogen (San Diego, CA, USA). The plasmid miniprep and gel extraction kits were purchased from Omega (Guangzhou, China). TRIzol and RT kits were obtained from TaKaRa (Dalian, China). Adenovirus shuttle plasmid pAdTrack-CMV, backbone plasmid pAdEasy-1 (present in *Escherichia coli* BJ5183), *E. coli* DH5α, human embryonic kidney cells (HEK293 cells), and the HT-29 colon cancer cell line (American Type Culture Collection, Manassas, VA, USA) were used from our lab.

### 2. Construction of Recombinant Adenovirus

The full-length DNA sequence of the *hITF* gene was amplified by polymerase chain reaction (PCR) using the plasmid containing the signal peptide and the mature peptide of *hITF* as a template, while the *Bgl*II and *Not*I cleavage points were introduced and then ligated with adenovirus shuttle plasmid pAdTrack-CMV by T4 DNA ligase after double digestion of *Bgl*II and *Not*I, which generates the complementary sticky ends with pAdTrack-CMV. The recombinant plasmid was transformed into *E. coli* DH5α and extracted for the double digestion test and following studies. After *Pme*I digestion, the linearized plasmid was in homologous recombination with the skeleton plasmid in BJ5183, while the positive clones were selected for culture, plasmid extraction, and *Pac*I digestion. After the recombinant adenoviral plasmid was digested by *Pac*I, the larger fragment was recovered using a gel extraction kit and transfected into HEK293 cells by a liposome. After 8 or 10 days, the recombinant adenovirus was collected by repeated freezing and thawing of the HEK293 cells and infected HEK293 cells; 2 or 3 days later, the cells showed the cytopathic effect (CPE) phenomenon and the virus was collected. The TCID50 method was used to calculate the adenovirus titer. The Karbers formula: The adenovirus titer = 10^1+1(positive−0.5)+1^ TCID50/mL = 10^1+1(positive−0.5)+1−0.7^pfu/mL.

### 3. Virus Infection of Colon Cancer Cells

HT-29 cells were cultured in Dulbecco’s modified Eagle medium (DMEM) containing 10% fetal calf serum and 100 U/mL penicillin and streptomycin in a 5% CO_2_ incubator at 37°C. The medium was changed every other day. The cells were subcultured every 3–4 days at a ratio of 1∶3, and those with good morphology were selected for further experiments. The HT-29 cells were seeded in 6-well plates at a density of 2×10^6^ cells per well and then placed in a 37°C incubator overnight to ensure good cell state and a moderate density of about 80% the next day. The required amount of virus vectors was calculated using a multiplicity of infection (MOI) of 50 after counting the digested cells per well. After the cells in the 6-well plates were washed with fresh culture medium twice, the virus was added and the cells were cultured in a 5% CO_2_ incubator at 37°C with fresh culture medium used to create a 2-mL volume in each well. Fluorescence expression was observed using confocal microscopy.

### 4. Reverse Transcription PCR (RT-PCR) Analysis

Cells were collected 2 days after the viral infection and total RNA was extracted and detected using RT-PCR. *β-actin* primers (internal control): upstream, 5′-TCGACAACGGCTCCGGCATG-3′; downstream, 5′-GCCAGGTCCAGACGCAGGAT-3′. The expected length of the PCR product was 514 bp.

Primers of the target *hITF* gene: upstream, 5′-GAGACTCGAGATGCTGGGGCTGGTCCTGG-3′; downstream, 5′-TTTAGAATTCGGAAGGTGCATTCTGCTTC-3′. The expected length of the PCR product was 240 bp.Amplification of *β-actin* was performed using the following protocol: initial denaturation at 94°C for 5 min followed by 30 cycles of denaturation at 94°C for 30 sec, annealing at 52°C for 30 sec, extension at 72°C for 30 sec, and a final extension at 72°C for 10 min.Amplification of *hITF* was performed using the following protocol: initial denaturation at 94°C for 5 min followed by 30 denaturation cycles at 94°C for 30 sec, annealing at 61°C for 30 sec, extension at 72°C for 30 sec, and a final extension at 72°C for 10 min.

### 5. Western Blotting

Serum-free medium was used to culture cells after viral infection for 6–8 h. The cells and supernatant were extracted 3 days later, the supernatant was precipitated and concentrated by trichloroacetic acid, and western blotting was performed after sodium dodecyl sulfate-polyacrylamide gel electrophoresis.

### 6. Immunofluorescence

HT-29 cells were seeded on glass coverslips and infected with Ad-hITF as described above. The cells were fixed for 20–30 min in a solution of 4% formaldehyde in phosphate buffered saline (PBS), washed in PBS, and permeabilized with 0.2% Triton X-100 for 3 min. The cells were then blocked for 20 min at room temperature using PBS supplemented with 5% bovine serum albumin followed by incubation with primary antibodies in PBS overnight at 4°C. Then slides were washed 3 times with PBS and incubated with secondary antibodies for 1 h at 37°C followed by 3 washes with PBS. DAPI was used to stain the nuclei and the slides were mounted with a glycerol-based mounting medium. All manipulations were conducted at room temperature, and secondary antibody incubation and mounting were performed in the dark. Slides were viewed and images were acquired on a Zeiss 510 Meta confocal microscope using LSM Image software (Zeiss).

### 7. Effects of Ad-hITF Infection on Colon Cancer Cell Migration

After collection, HT-29 cells in the logarithm phase were seeded in a 24-well plate adjusted to 5×10^5^ cells per well and cultured at 37°C in a 5% CO_2_ incubator overnight. A virus with an MOI of 50 was added to the cells and cultured at 37°C in 5% CO_2_ for 2–3 days. After all of the cells expressed GFP, a straight line was streaked at the bottom of the 24-well plate with a 10-µL tip, resulting in linear mechanical damage approximately 300 µm wide. The cells were washed twice with serum-free medium to remove cell debris. Twenty-four hours after the addition of serum-free DMEM, cell migration was observed and the difference in scratch width was measured.

### 8. *In vivo* Experiments

#### 8.1 Preparation and grouping of the mice model after burning

Healthy male and female Kunming adult mice weighing 20±2 g were randomly divided into 3 groups: normal (C), burn (B) and gene therapy with recombinant adenovirus (Ad-hITF). After fasting for 12 h, the mice were anesthetized with 1% pentobarbital sodium (40 mg/kg) by intraperitoneal injection, and the fur on their backs was shaved with an electric shaver and removed using 8% sodium sulfide. After the mice were burned with 3% solid gasoline for 12 sec, 50 mL/kg Ringer's lactate was immediately injected intraperitoneally for anti-shock. The mice were given special animal feed and free access to water [5].

#### 8.2 Phase setting and administration dosage

The experiment were divided into 4 phases, post-burn days (PBD) 1, 3, 5, and 7 with B group and Ad-hITF group in every phase and at least 6 mice from the B and Ad-hITF groups in each phase. Mice in the Ad-hITF group were gavaged with the first recombinant adenovirus 2 days before the injury and the second adenovirus after post-anesthesia recovery with the same dose of 2×10^8^ pfu/mouse [11], while B group was gavaged twice with physiological saline with the same amount at the same time.

#### 8.3 Intestinal mucosa morphology

After the mice were sacrificed, the intestines were removed, cut along the longitudinal axis, and then flattened after isotonic saline rinsing. Mucosal damage was observed under a magnifier and the injury index was scored according to the Colonic Mucosa Damage Index (CMDI) standard: 0, no damage; 1, mild hyperemia, edema, smooth surface, and no erosion or ulceration; 2, hyperemia, edema, rough and granular mucosa, and erosion or intestinal adhesion; 3, severe hyperemia and edema, necrosis and ulceration on the mucosal surface with the largest vertical diameter <1.0 cm as well as thickening wall or necrosis and inflammation on the surface; and 4, the maximum longitudinal diameter of ulceration >1.0 cm or intestinal necrosis [12].

#### 8.4 Intestinal histopathology

Intestinal tissues were fixed in 10% formalin solution, embedded by paraffin, and then observed and photographed by microscopy after hematoxylin and eosin (HE) staining.

### 9. Statistical Analysis

Statistical evaluation was performed using SPSS13.0 (SPSS,Chicago, IL, USA). Data are expressed mean±SD. The results were compared by using one-way analysis of variance (ANOVA) followed by Scheffe’s post hoc test for multiple comparisons. A value of P<0.05 was considered statistically significant.

## Results

### 1. Construction of Recombinant Adenovirus

The target gene and adenovirus shuttle plasmid pAdTrack-CMV were double-digested and ligated overnight and then transformed into *E. coli* DH5α competent cells. Plasmids were extracted from 2 randomly selected colonies, and a fragment approximately 996 bp long appeared after double-digestion of *Bgl*II and *Not*I confirmed positivity. After homologous recombination of the recombinant shuttle plasmid and backbone plasmid, dozens of large, medium and small colonies appeared. A number of the small and medium colonies were randomly selected for bacterial culture, plasmid extraction and PacI digestion for verification. In this study, a fragment approximately 3.0 kb long appeared after digestion, confirming successful recombination of the Ad-hITF adenovirus plasmid. Ten days after Ad-hITF infection of the HEK293 cells by Lipofectamine 2000, all of the cells turned green, a large number showed typical CPE such as swelling and roundness, some cells appeared like a bunch of grapes, and some cells were floating and round under light microscopy. Three days after infection with the packaged viral supernatant, almost all of the HEK293 cells turned green with high fluorescence intensity, and a large number of cells became round and floated. The adenovirus titer after amplification = 10^1+1 (1+1+1+1+1+1+1+1+0.5+0−0.5)^ TCID 50/mL = 10^10^ TCID 50/mL or = 10^9.3^pfu/mL = 2×10^9^pfu/mL (calculated using the Karber formula).

### 2. Infection of HT-29 Cell by Recombinant Ad-hITF

**Figure 1:**
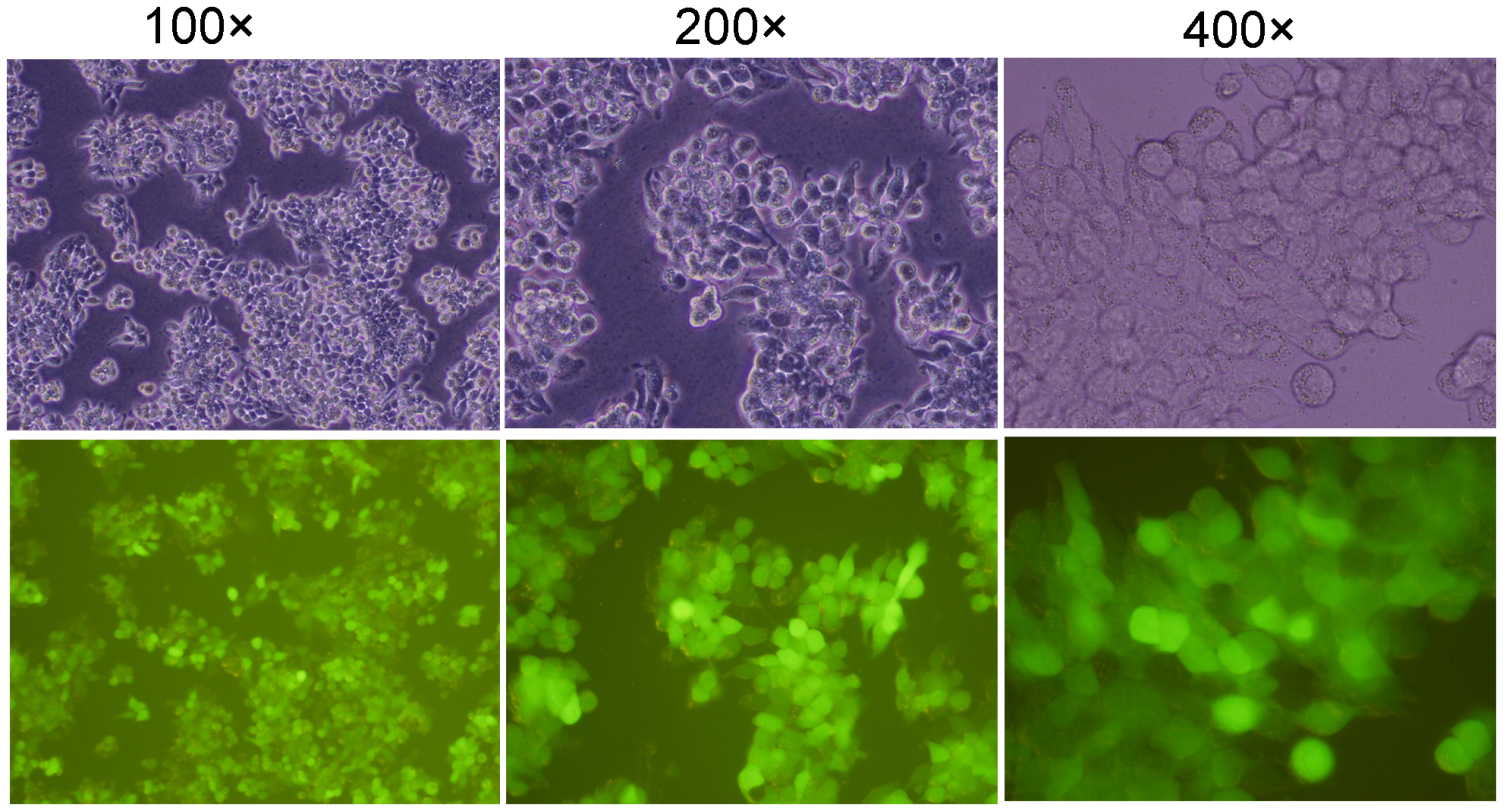
Infection of HT-29 cells with recombinant Ad-hITF.

HT-29 cells were infected by Ad-hITF at an MOI of 50. The efficiency was nearly 100% by roughly estimation (the proportion of cells with GFP expression in a random vision), but cell morphology did not change under light microscopy (Fig. 1).

### 3. RT-PCR Analysis

**Figure 2:**
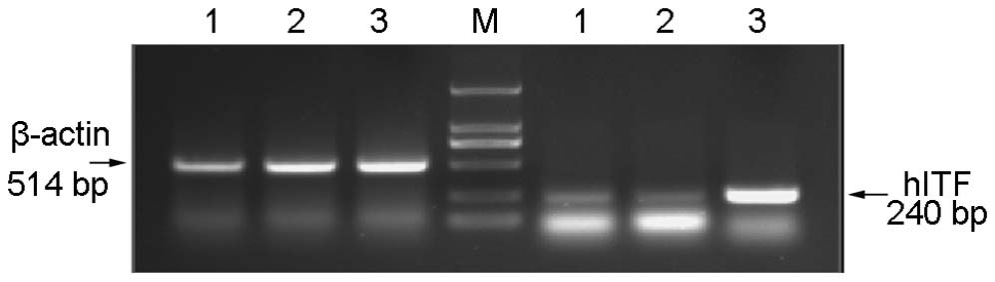
Agarose gel (1.2%) electrophoresis of the reverse transcription-polymerase chain reaction (RT-PCR) product. HT-29 cells were infected with the recombinant Ad-hITF adenovirus or the empty Ad-EGFP virus. Two days after viral infection, total RNA was extracted and detected using RT-PCR. Lane 1, HT-29 cells; Lane 2, infection with Ad-EGFP; Lane 3, infection with Ad-hITF; Lane M, DL2000 marker.

Based on RT-PCR analysis, the target band was amplified in HT-29 cells after Ad-hITF infection, while no band was seen in those with no or empty viral infection, indicating transcription of the *hITF* gene into HT-29 cells after Ad-hITF infection (Fig. 2).

### 4. Western Blot Analysis

**Figure 3:**
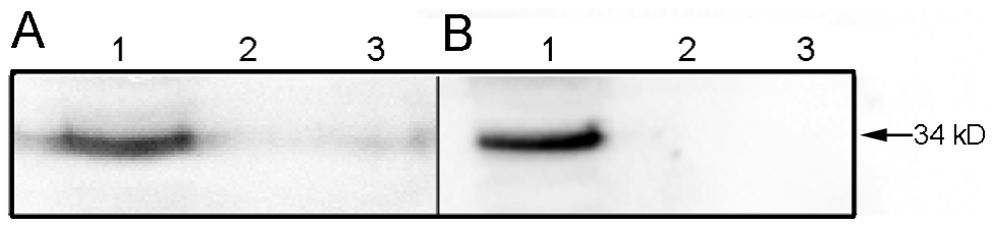
Western blot analysis of hITF. After infection of HT-29 cells by the adenovirus, western blot analysis of hITF in the cells (A) or culture supernatant (B) was performed. Lane 1, infection with Ad-hITF; Lane 2, infection with Ad-EGFP; Lane 3, HT-29 cells.

Western blot results showed that an obvious band appeared in the cells and the supernatant after Ad-hITF infection while no band appears in others, indicating that the expression products can recognize anti-hITF monoclonal antibodies and possess good antigenicity (Fig. 3).

### 5. Immunofluorescence Analysis

**Figure 4:**
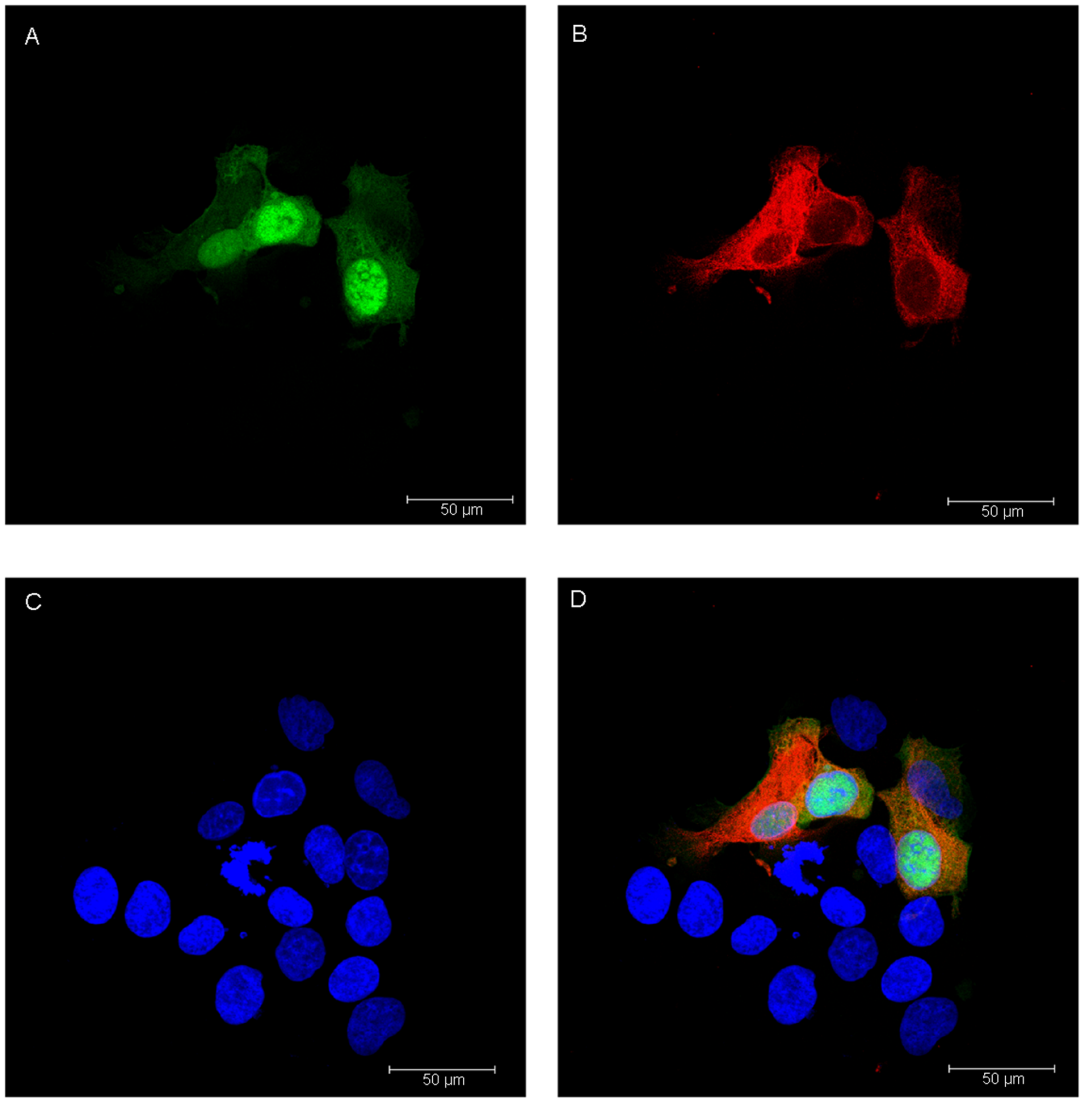
Fluorescent images of HT-29 cells infected with Ad-hITF. The hITF was detected using mouse anti-hITF antibodies and goat anti-mouse TRITC-labeled secondary antibodies. Nuclei were stained with DAPI. A, GFP; B, hITF; C, cell nuclei; D, merged images.

As shown above, Ad-hITF can infect colon cancer cells. Figure 4(A) shows that GFP can be expressed in the nucleus and cytoplasm, but mainly in the nucleus. Figure 4(B) shows that exogenous hITF in red color can be expressed in the nucleus and cytoplasm but is mainly expressed in the cytoplasm. Figure 4(C) shows the blue nucleus after DAPI staining, while Figure 4(D) shows the merged image.

### 6. Effect of Ad-hITF on Colon Cancer Cell Migration

**Figure 5:**
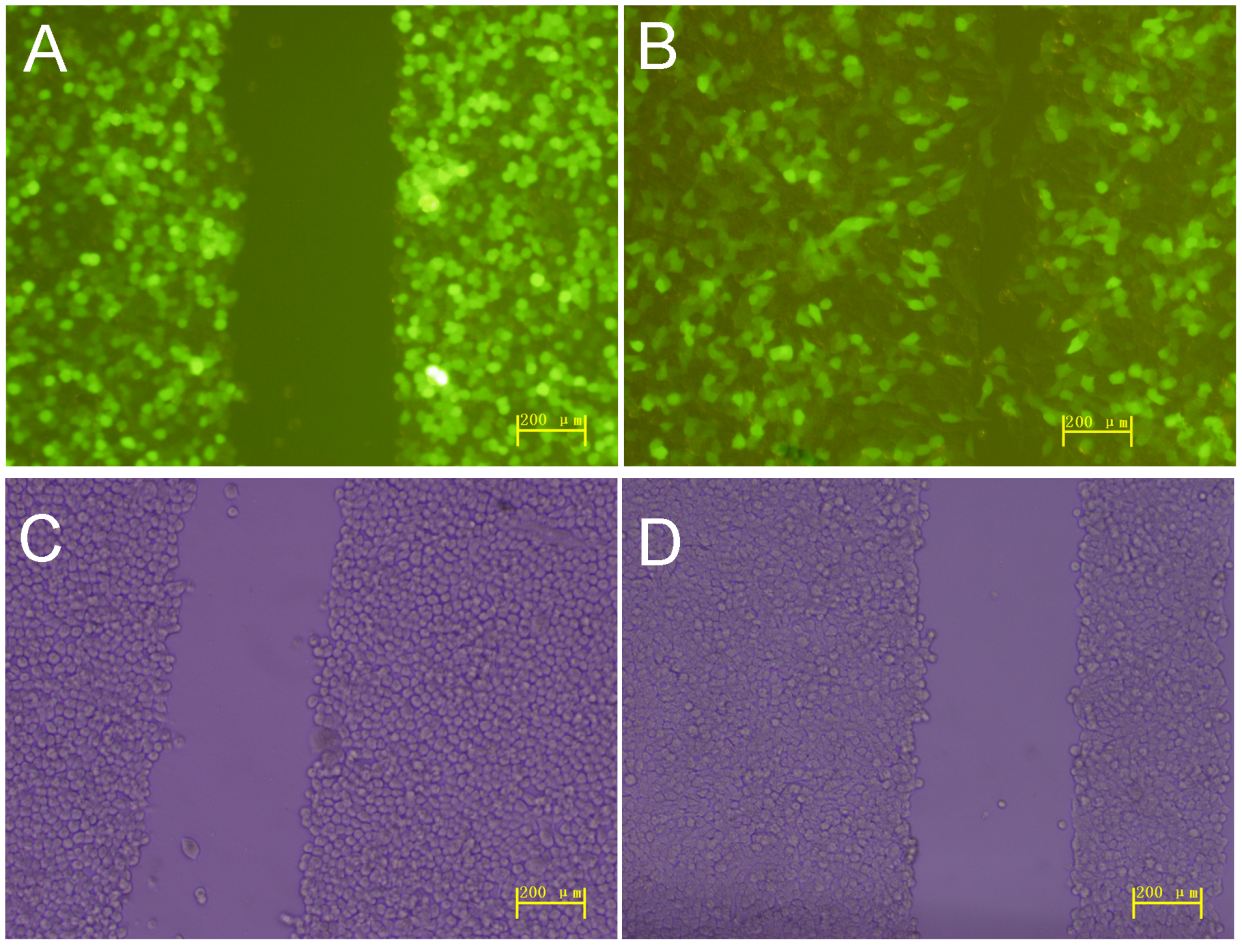
Effect of Ad-hITF on cell migration. HT-29 cells were infected with recombinant Ad-hITF adenovirus (A) or empty Ad-EGFP virus (C). Wounds were established in confluent monolayers of HT-29 cells and the wounded monolayers infected by recombinant Ad-hITF adenovirus (B) or empty Ad-EGFP virus (D) were cultured for 24 h in serum-free Dulbecco’s modified Eagle medium.

As shown in Figure 5, the scratches were approximately 300 µm wide and were significantly reduced after Ad-hITF infection of HT-29 cells after 24 h (P<0.01), while no significant difference was seen after the empty Ad-EGFP viral infection.

### 7. Effect of Ad-hITF on the CMDI

Intestinal mucosa injury appeared as early pathogenesis, heavy damage, and obvious pathological changes one day after the burn, while the CMDI was significantly higher than that of the pre-injury and then became higher and peaked 3 days after the burn (2.71±0.49). After that, the damage was gradually repaired and the CMDI showed a downward trend and became normal 7 days after the injury. Compared with the burn group, the CMDI in the Ad-hITF group after the injury was significantly lower, especially on PBD 3–5, a difference that was statistically significant (1.67±0.52 vs. 2.71±0.49,0.88±0.35 vs. 1.57±0.53; P<0.01) (Table 1).

**Table 1:** Effect of Ad-hITF on the Colonic Mucosa Damage Index of the intestinal mucosa (mean ± SD).

	PBD1	PBD3	PBD5	PBD7
C	0.00±0.00 (6)			
B	2.63±0.52 (8)**	2.71±0.49 (7)**	1.57±0.53 (7)**	0.83±0.41 (6)**
Ad-hITF	2.43±0.53 (7)**	1.67±0.52(6)**^ΔΔ^	0.88±0.35(8)**^ΔΔ^	0.50±0.55 (6)

PBD, post-burn day.

Numbers in parentheses represent the number of mice in each group.

Compared with C, **p<0.01; compared with B, ^ΔΔ^p<0.01.

All statistics were performed using one-way ANOVA with Scheffe’s post hoc test.

### 8. Pathological Changes of Intestinal Mucosa

**Figure 6:**
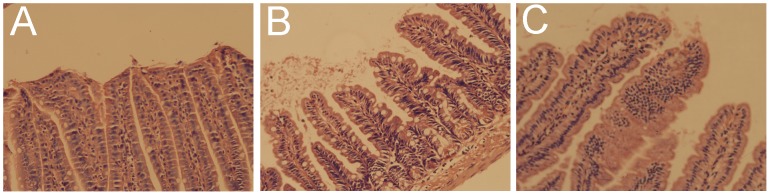
The therapeutic effect of Ad-hITF on burn-induced intestinal lesions. Ad-hITF or saline was given orally to the mice inflicted with thermal injury. The mice were sacrificed on PBD3. (A) Normal intestinal mucosa in the C group (hematoxylin and eosin [HE], ×200). (B) Representative mouse intestine treated with saline. Note that extensive necrotic lesions can be seen in the corpus. Diffusely severe hemorrhagic and necrotic mucosa were confirmed using histological examination (HE, ×200). (C) Representative mouse intestine in animals receiving Ad-hITF therapy. No necrosis is noted except for diffuse edema; microscopic examination demonstrated that the superficial epithelium was intact (HE, ×200).

As shown above, intestinal histopathology revealed that the intestinal villi were arranged in neat rows, the epithelial cell layer was intact with a smooth surface, and a thin layer of mucus was seen on the mucosa in the normal mouse model (Fig. 6A). In the burn group, the intestinal villi were disorderly arranged and fractured and the epithelial cells were edematous, denatured, necrotic, and even partially deciduous, leaving the laminae propria bare (Fig. 6B). In the Ad-hITF group, the pathological changes mentioned above were alleviated and only congestion and edema of the epithelial cells and mild expansion of the central lacteal were seen, and the intestinal epithelium remained intact with no large area of hemorrhage, necrosis, or shedding (Fig. 6C).

## Discussion

ITF, a small polypeptide with potential medical value, alleviates the gastrointestinal mucosal injury caused by various injury factors and promotes the repair of damaged mucosa. Full-length *hITF* cDNA contains 222-bp DNA (GenBank Accession No. NM003226) that encodes 73 amino acids including the first 14-aa signal peptide followed by a 59-aa mature peptide [13], [14]. Precursor hITF is synthesized in intestinal goblet cells and travels to the gut after being secreted under signal peptide command. Meanwhile, mature hITF forms after the cleavage of signal peptide. Our study focused on the full-length hITF that plays a physiological role after being secreted into the intestine. ITF is thought to play an important role in protecting the gastrointestinal mucosa [15], but its low yield limits its practical use. Gene therapy is a newly developed technology for the treatment of diseases by delivering genes into patients and a new “molecular therapy” accompanied by the development of genetic engineering and molecular biology [6], [7]. If ITF can be delivered into the human body by gene therapy and abundantly expressed *in vivo*, it will both simplify the tedious preparation procedure and reduce production costs. In this study, the full-length *hITF* was delivered into the gastrointestinal tract by gene therapy and stably expressed in the gastrointestinal mucosa to compensate for the source insufficiency and achieve the same effects as the exogenous protein.

Compared with other gene delivery carrier types, the advantages of the third-generation adenovirus used in our work include a wide host range, high infection rate, high expression levels of exogenous gene, and so on, which makes it favored by many scholars [9], [10]. First, the recombinant shuttle plasmid pAdTrack-CMV-hITF was constructed by *hITF* gene ligation with a shuttle plasmid (pAdTrack-CMV) containing the GFP reporter gene to facilitate detection and observation. The linearized recombinant shuttle plasmid was in homologous recombination with backbone plasmid pAdEasy-1 in the *E. coli* BJ5183 strain, constructing the recombinant Ad-hITF adenoviral vector. Using the newly constructed AdEasy™ system in our study, the adenovirus shuttle plasmid containing the exogenous gene was infected into an *E. coli* strain containing the adenoviral backbone plasmid. After homologous recombination by Cre recombinase and linearization, the recombinant adenovirus vector was infected into the 293 cells in which the recombinant virus particles were packaged [16]. All of the procedures above were finished in *E. coli*. The relatively simple and highly efficient operation and >60% homologous recombination rate recently led to wider application [17].

Human intestinal epithelial cells are subjected to daily mechanical friction and stimulated substances such as acids, alkalines, food, and even bacteria; thus, it is essential that cell adhesion be very tight to prevent infection. In our experiments, after infected with an MOI of 50 (earlier studies), almost all of the cells expressed GFP, indicating that the recombinant adenovirus infection rate was nearly 100% and the cell morphology was the same as normal cells without changes in growth rate and adhesion based on fluorescence microscopy. Based on RT-PCR and western blotting analysis, the hITF was transcribed, translated by the colon cells after infection, and secreted into the medium, whereas no expression was seen in the cells with no or empty carrier infection. Thereafter, immunofluorescence analysis showed that the hITF was distributed mainly within the cytoplasm and little was seen in the nucleus after recombinant adenovirus infection of colon cancer cells, indicating that the hITF was mainly synthesized in the endoplasmic reticulum of the cytoplasm and neither entered nor functioned within the nucleus.

Compared with other organs, gastrointestinal mucosa is easier to damage because it connects with the outside and is usually invaded by harmful substances. Therefore, its long-term evolution resulted in a perfect self-protective mechanism. The repair of gastrointestinal mucosa depends on cell proliferation and migration; in particular, a quick repair mechanism of cell migration has great significance in its protection [18], [19]. Cell migration depends on continuous division, proliferation and migration of crypt cells to lesions to replace damaged cells. However, whether *hITF* gene therapy can promote intestinal epithelial cell migration is unknown. To verify its physiological role, we performed mechanical scratches when the cells were grown to full integration after recombinant adenovirus infection of colon cancer cells and used cells with empty viral infection as a control. The results showed that colon cancer cell migration was significantly greatly after infection of the recombinant adenovirus. The process of ITF-promoted gastrointestinal mucosal repair was as follows: after injury (such as mechanical injury), intestinal epithelial cell adhesion is interrupted and the fast phase of intestinal repair starts, ITF expression is increased within a few minutes, and increasing amounts of ITF promote rapid migration of normal epithelial cells to the injury site to replace the injured cells. The specific mechanism is unknown but may be as follows. First, expression of the epithelial cell adhesion molecule - cadherin (E-cadherin) and its cross-linking with the cell membrane may be inhibited. ITF may affect E-cadherin expression and localization and induce tyrosine phosphorylation of catenin, which causes loss of cell adhesion, further impedes tight cellular adhesion, and promotes epithelial cell migration [20], [21]. Under ITF stimulation, the Vangl1 protein may be Ser/Thr phosphorylated, the cross-linking of cell membrane and E-cadherin may disappear, and cell migration may be accelerated once mucosal repair begins [22].To verify the effect of *hITF* gene therapy, after preparing mice model with severe burns, we gavaged them with recombinant adenovirus and then detected the specimens at specific points in time. The CMDI judges injury severity [23]. Our study results showed that intestinal mucosa injury appeared early in pathogenesis and demonstrated heavy damage and obvious pathological changes one day after the burn, while the post-injury CMDI was significantly higher than the pre-injury CMDI, became worse, and peaked 3 days after the burn. Although the CMDI of the Ad-hITF group was significantly higher than that of the C group, it was significantly lower than that of the B group; these differences were statistically significant, especially 3 days after the burn. The CMDI of the Ad-hITF group returned to normal 7 days after the injury, earlier than that of the B group. The mouse model 3 days after injury, which had the highest CMDI, was chosen for further histopathology studies. In the burn group, the intestinal villi at this stage were in disarray and fractured, and the epithelial cells were edematous, denatured, necrotic, and even partially deciduous, resulting in exposed laminae propria. The Ad-hITF group demonstrated slight histological changes and intact intestinal epithelium with no large areas of hemorrhage, necrosis, or shedding.

In summary, we constructed a stable and efficient recombinant adenovirus carrier containing the *hITF* gene, and *in vitro* analysis showed that the virus could efficiently infect colon cancer cells, secrete and express hITF, and significantly accelerate cell migration. *In vivo* analysis demonstrated that *hITF* gene therapy could significantly alleviate intestinal mucosa injury after a burn and accelerate reconstruction of the damaged mucosal barrier. The current work has laid the foundation for *hITF* gene therapy.
